# I Teach Nursing as a Male Nursing Educator: The East Asian Perspective, Context, and Social Cognitive Career Experiences

**DOI:** 10.3390/ijerph17124327

**Published:** 2020-06-17

**Authors:** Luis Miguel Dos Santos

**Affiliations:** Woosong Language Institute, Woosong University, Daejeon 34514, Korea; luismigueldossantos@yahoo.com; Tel.: +82-010-3066-7818

**Keywords:** Asian perspective, male nurse, nursing education, male nursing educator, nursing shortage, social bias, social cognitive career theory

## Abstract

Social and cultural backgrounds, as well as understanding, play key roles in workforce development and human resource shortages, which are associated with the transition to nursing education and teaching from frontline nursing practices. A qualitative method, with the direction of the general inductive approach, was employed in this study. The researcher collected information from 18 male nursing educators who switched their senior roles (from the frontline and practicing fields to nursing education) at nursing schools in South Korea, Japan, Taiwan, China, and Malaysia. Three interview sessions were used to collect information. Three themes were merged from the information: (i) gender-oriented knowledge, teaching and learning; (ii) respect; and (iii) health promotion. More importantly, participants advocated that their male roles and identities provided uniqueness to patients, students, parents, and the general public concerning Asian customs and practices. Based on the social cognitive career theory, personal goals and achievements of career satisfaction took important roles. Although the general public may not agree with these career decisions, due to gender and social biases, participants continued to contribute their energy and knowledge in the health and social caring professions.

## 1. Introduction

Nursing is one of the professions that has faced significant human resource shortages and turnover rates for nearly a century. Although many government agencies, schools, and NGOs provide traditional and alternative teaching and training options for both traditionally aged and career-changing nurses, these human resource shortages are not changing. Providing nursing education and lessons is difficult due to social and cultural issues and various background expectations, particularly for male nursing educators and educators with different backgrounds who instruct nursing students [1]. Although teaching nursing education and health sciences to young college and university students may be difficult and require sensitivity, nursing educators should still provide appropriate instructions and knowledge. However, due to cultural differences, gender issues, expectations, industries’ concerns, and even language barriers, a large number of nursing and health sciences educators leave their positions within the first few years of teaching [2]. Although these nursing educators leave their teaching professions, many return to the frontline as registered nurses or professional nursing leaders. 

Teaching nursing education and health sciences in the East Asian region is an attractive occupation and form of career development for teachers, particularly teachers at vocational schools, community colleges, and universities, after completing university and postgraduate degrees [3]. For more than 20 years, the East Asian region has been a popular destination for nursing and health sciences educators to start their teaching careers [4,5]. Due to the rapid economic and cultural developments that have taken place in East Asia, the region attracts a large number of international professionals starting their career pathways, particularly nursing and health sciences educators. 

However, even though nursing schools recruit nursing educators and training professionals every year, the turnover rate in this field is still significant, particularly for male nursing educators, who have unique experiences to offer that female nurses cannot provide. Unlike other westernized societies, due to social, cultural, and religious practices and expectations, the responsibilities of male and female nursing educators and nursing practitioners are separated. Therefore, recruiting male nursing educators from a smaller population is even harder. As a result, nursing schools spend a significant amount of resources and training on nursing educators and health sciences educators who do not have any teaching experience [6].

The high turnover rates of nursing and health sciences educators usually negatively impact the operation and overall performance of nursing students, in-service nurses, and even patients. Sometimes, nursing school professionals find that overall workplace morale is influenced by the turnover rate of professional co-workers [7]. For more than two decades, several studies [8] have indicated that nursing school administrators might not be able to replace top-tier and qualified nursing and health sciences educators and professional staff due to the significant annual turnover rate [6]. Although a reasonable amount of turnover always happens in contemporary organizations and schools, unreasonable turnover frequencies hurt nursing school reputations and student learning experiences [9]. In short, there are four different perspectives that are harmful in regard to these departures: the financial perspective, the human resources perspective, student perspective, and the hospital and employer perspective.

First, the researcher examined the financial perspective. Nursing school administrators expend a significant amount of resources to host recruitment fairs and pay international agent fees for qualified nursing and health sciences educators, as well as in the course of hiring professional staff. These expenses are unavoidable due to school developments and expansions. However, these financial resources must also be allocated to teachers’ professional development, student learning experiences, and even salary increases [7].

Second, the researcher examined the human resources perspective. Nursing school administrators and leaders need to spend time, resources, and efforts on professional training and development, to train incoming nursing and health sciences educators and professional school staff, such as in regard to the usage of computer systems, classroom management for both local and international students, languages, and teaching strategies [10]. Although nursing schools may allocate teachers’ professional hours for forms of training, such as these during each semester or term, mid-level and senior-level nursing and health sciences educators in the same nursing school could become confused due to repeated information [11].

Third, the researcher examined the student perspective. Many nursing schools enroll students from different parts of the world, with various backgrounds, perspectives, understandings, customs, and languages. More than half of the schools’ student populations usually consist of students from different cultural backgrounds. In the East Asian context, most students tend to advocate the cohort learning model, in which a leading teacher and a student counselor take care of student educations throughout the years they attend nursing school [12]. However, the frequent departure of teachers and professional school staff may harm the relationships formed between students and educators, as new staff do not understand, at first, the student populations as well as their predecessors. The student populations equally do not understand new staff as well; therefore, they experience a lapse in understanding of how to work with teacher expectations [13].

Fourth, the researcher examined the hospital and employer perspective. Although turnover rates do not directly impact the benefits of the hospital and the employer, potential employers still need to share information with new teachers and professional school staff each year, to update their potential employees’ working knowledge and improve their overall performance. However, if staff can follow their nursing students’ educations consistently, solid relationships and confidence will be built up [14]. Frequent departures may increase uncertainty in these respects.

In short, although reasonable turnover rates may increase the overall performance of nursing schools and improve student experience, many studies indicate that teachers and professional school staff leaving negatively impacts student performance and learning experience [15], particularly in the fields of nursing and health sciences education. The literature suggests that such frequent departures increase concerns in regard to the financial perspective, the human resources perspective, student perspective, and hospital and employer perspective.

### 1.1. Purpose of the Study 

There are two purposes for this research study. First, although the current literature database contains a great deal of research and a number of projects about nurses and nursing educator turnover, career decisions, and professional development in a school setting in the East Asian region, most of these studies focus on administrative decisions, management, leadership, teaching management, and teaching strategies. Only a few researchers and studies have focused on the directions of nursing and health sciences educator career decisions and career development pathways in regard to nursing schools, particularly for male nursing educators [16]. Therefore, the results of this study can help to outline the career decisions, career development pathways, and professional development of the nursing school and nursing and health sciences educators who decide to invest their lifelong career development in nursing school settings in the East Asian region, particularly male nursing educators [17].

Second, nursing school administrators and leaders are generally concerned about how to retain their teachers and professional staff, as turnover rates cost time and resources in terms of the additional training and recruitment that become necessary as a result, particularly in the fields of nursing and health sciences education [18]. Therefore, seeking understanding, feedback, and sharing from nursing educators are meaningful. With the focus on the East Asian region, the results of this research study may outline how to increase the morale and confidence of male educators in nursing school settings [19].

The research study was guided by the following research questions:

- Why do participants decide to invest their lifelong career development in a nursing school setting in the East Asian region as male nursing educators?
- How do participants describe their roles as male nursing educators in a nursing school setting in the East Asian region?

### 1.2. Theoretical Framework

In order to examine the background, and gain a holistic picture of male nursing education and health educators, regarding the career decision and career development at the nursing school setting in the East Asian region, the Social Cognitive Career Theory (SCCT) has been employed for the research [20,21,22,23,24]. The SCCT aimed to understand, explore, and discover the career decision of academic and career choice chances, and the performance and limitation in individual education and career development goals [16,25,26,27,28,29,30,31].

Under guidelines from the social cognitive theory, researchers developed the Social Cognitive Career Theory with the perspective of career decision and development, focusing on individuals, behaviors, and environmental factors. Both Bandura [32] and Lent et al. [20] advocated that individual behaviors are not an aftermath of interactive behaviors and movements between individuals and the environmental (external) factors, but rather, behaviors and movements serve as interactive elements and considerations by impacting the aftermath, thereby, impacting the thinking, internal elements, emotions, and follow-up movements and activities of individuals [16,25,26,27,28,29,30,31].

The Social Cognitive Career Theory [16,25,26,27,28,29,30,31] has three essential points for its modeling in the areas of career decision and development: (i) the formation and elaboration of career-related interests, (ii) the election of academic and career choice options, and (iii) the performance and persistence in educational and occupational pursuits [20,24,33]. It is worth noting that the advocators of the Social Cognitive Career Theory argued that the theory groups the differences between the intentions (people’s personal beliefs, planned goals, and internal demands) and behaviors (exercises, movements, conducts, practices, and behaviors) as individuals like to conduct what they always believe. In other words, individuals tend to conduct and exercise their career decisions and developments based on what they believe and advocate instead of arranged planning [16,25,26,27,28,29,30,31]. As a result, the Social Cognitive Career Theory [16,25,26,27,28,29,30,31] expands the relationships and bonds among how cultural, social, and financial factors and considerations may influence individual self-knowledge and career decisions and development.

## 2. Materials and Methods 

The researcher decided to employ the qualitative research method [34,35,36] to collect and analyze data information into themes and subthemes for reporting. The nature of this study is to understand and explore the lived stories and experiences from the participants and their inner social worlds. Without in-depth interview sessions, to each, the researcher could not capture the life experiences from the participants.

### 2.1. Participants

A total of 18 male nursing educators, with both administrative and frontline experiences at hospital and nursing school environments, were invited. All agreed to participate in this study. Due to the nature of nursing educators with rich experience in the teaching profession, all had at least 20 years of both frontline and teaching experience.

The purposive sampling strategy [12,16,31,35,36,37] was employed to recruit the participants. First, the researcher searched the database of nursing educators in South Korea, Japan, Taiwan, China, and Malaysia. Second, the researcher sent an email invitation to the nursing school administrators for the criteria of invitation, such as years of experience. Third, the administrators responded to the researcher about the results of personnel in their school and forwarded the invitation to each potential participant. Fourth, as a result, the following 18 participants matched the criteria for the research study and agreed to participate.

Due to the concerns of privacy, the researcher arranged a pseudonym to each participant in order to mask their identity to other people, particularly their employers and potential readers in the field. As the names of the employers, hospitals, nursing schools, and departments that the participants used to work did not take any role in the study, the researcher decided to mask all of this information [35,36]. Table 1 refers to the demography of the participants. 

### 2.2. Data Collection 

The in-depth, open-ended, and semi-structured interview tool [35,36] was employed to collect meaningful data information from the participants. The individuals shared their understanding, life experience, sense-making process, lived stories, and feedback as male nursing educators in the East Asian region. All participants have voluntarily participated in this study. The general inductive approach [38] was employed for qualitative data collection and analysis [39]. Appendix A listed the interview questions. It is worth noting that the interview questions were developed based on the SCCT and the objectives of the study (i.e., the career perspectives and experiences of career development). 

In order to seek rich data information from the participants, the researcher invited each for three interview sessions. According to Seidman [40,41], a relationship should be built between the participant and researcher in order to capture the real information. Although the researcher did not know each before the interview, three-round interview sessions increased the relationship between each other. As a result, all confirmed the interview information was true for reporting. Each interview lasted between 60 to 89 minutes. In addition to these interview sessions, in order to confirm the validity of the data information, the researcher sent the related transcript to each participant for confirmation. With a brief member checking interview session, all participants agreed their sharing for further reporting. 

Not all participants were native English speakers. As the researcher is multilingual in English, Chinese Mandarin, Chinese Cantonese, and Japanese, participants were able to share some of their ideas in the abovementioned language. Moreover, participants might have requested an interpreter for immediate sharing. Further, if the participants shared information other than English during the interview sessions without the interpreter, the researcher translated the conversation and written transcripts with the coordination of a professional translator. As a result, only one Korean participant shared several parts in the Korean language. All of the rest shared the English language conversation.

### 2.3. Data Analysis 

After the data collection procedure, large-size written transcripts were created based on in-depth, open-ended, and semi-structured interviews [16]. Qualitative researchers [35,36,37,39,42] advocated that large-size data information should be reduced and narrowed down into categories and themes for reporting. Therefore, the researcher followed the recommendation of an open-coding strategy to narrow down the information into first-level themes and subthemes. In fact, the open-coding strategy always allows the researcher to gather and narrow the data information from 54 interview sessions. After the open-coding procedure, the researcher categorized 20 themes and 34 subthemes for the first-level categories and groups. 

However, the first-level themes and subthemes were too large for reporting. Therefore, the axial-coding technique must be employed to narrow down the information. Based on the first-level themes and subthemes, the researcher categorized the patterns and groups into the second-level themes and subthemes. After several rounds of data analysis, three themes and six subthemes were merged. The following session will explain the themes and subthemes into the table format.

### 2.4. Human Subject Protection 

All of the signed and unsigned agreements, personal contact, audio recording, written transcripts, computer, and related materials were locked in a password-protected cabinet. Only the researcher had the means to open it. After the study was completed, the researcher immediately destroyed and deleted all related materials for personal privacy. 

Due to the content forms and agreements, the place of origin, religion, university employment, and age were masked due to privacy. Due to the small population and closed professional network, particularly for male nursing educators in the East Asian region, the researcher needed to protect the information of the participants. All subjects gave their informed consent for inclusion before they participated in the study. The study was conducted in accordance with the Declaration of Helsinki, and the protocol was approved by the University Ethics Committee of the Social Caring Project with (2019/2020/08-12). 

## 3. Results and Discussions

During interview sessions, participants answered the same open-ended and semi-structured interview protocol and related interview questions [34,36,39] about why they decided to invest their lifelong career development in nursing school settings in the East Asian region as male nursing educators. They were also asked to describe their roles as male nursing educators in nursing school settings in the East Asian region. Although all of the participants were experienced, and senior-level nursing educators with similar professional backgrounds and experience in hospitals and school environments, their lived stories and personal backgrounds were different. Social and cultural backgrounds in the East Asian region are unique [43,44]. Therefore, the results highlighted a number of social and cultural expectations of the traditional Eastern thinking and ideas, including male-oriented knowledge, respect, and the promotion of health, based on male perspectives and conceptions. Table 2 refers to the themes and subthemes of this study. 

### 3.1. Gendered-Oriented Knowledge, Teaching, and Learning 

For centuries, nursing has been viewed as a female-dominated profession, and most registered nurses and nursing practitioners are women [6]. However, this does not mean that the profession has rejected male individuals in terms of career development and services, including nursing education and training. Although nursing is a female-dominated occupation, male human resources and personnel are necessary due to male-oriented treatments and care that must be provided for male patients and male-centered work [45]. For example, many participants expressed the way in which male patients require male nurses and doctors for their treatment and care due to social and cultural expectations. One said:

*In some religious practices, male patients must be treated by other male medical professionals. Also, for some treatments, such as bladder catheters for male patients, male nurses and medical professionals are needed due to requests from the patients. Without enough male professionals, it is hard to balance the demands*.

(Participant #16)

#### 3.1.1. Male-Oriented Knowledge and Skills in Operation Management

In many nursing schools and nursing environments, female individuals hold most of the positions and roles, due to imbalanced gender issues and social bias toward the occupation. In other words, due to the significant and increasing number of female nursing professionals in the field, the number of male nurses is significantly low. Based on differences in the social roles men and women are expected to play, there can be differences between male and female personalities and professional development. Male individuals are perceived as being logical, whereas female individuals are often expected to focus on honing their communicative skills within interpersonal interactions [46,47,48,49,50,51,52,53]. Some participants argued that each department and sector should employ a balanced number of male and female employees and professionals in order to arrange work responsibilities based on the abilities of different individuals. Although many professions recruit a larger number of male individuals into their systems, due to gender biases and discrimination, nursing professions face similar problems in regard to male individuals. For example, some participants suggested that nursing schools and nursing departments should train and provide opportunities to male nurses in regard to appropriate administrative positions and operational positions, due to their advantages and personalities. One participant said:

*Male nurses usually have a better level of logical thinking. In contrast, female nurses usually have better skills in communication and public relations. We should arrange and appoint the right people in the right positions. In the postgraduate programmes in nursing at my school, we need to provide such thinking, ideas, and managerial styles to nursing leaders, to reform their managerial styles at the hospital*.

(Participant #3)

Family responsibilities and roles are another consideration concerning why male nurses should be trained in nursing professions. Some participants suggested that female nurses and practitioners need to balance time between work and family, more so than their male counterparts, due to the social role they are expected to play of caretakers for their family members. Although male professionals should spend an equal amount of time as female professionals on family responsibilities, female professionals tend to contribute additional resources and energy to their families, due to the expected gender roles they have to play in East Asian societies [6,10,18,54,55,56,57,58]. Based on this characteristic, all participants shared similar ideas and feedback about female professionals and their family responsibilities during the interview sessions. One said:

*Female nurses and mothers put family first; their jobs can be their third or the fourth priority, but male individuals should balance this out, as they are often the main resource providers of the family. Male nurses can contribute more to their positions and development. Male nurses are also interested in building their career development*.

(Participant #6)

Some participants also shared their views that male nurses may be more willing to work overnight shifts than female nurses, due to family responsibilities. Many suggested that the mother’s role is more vital to a child’s development than the father’s role. As many nurses are female professionals, male individuals are willing to arrange spaces for other female professionals, particularly co-workers with family responsibilities. One participant said:

*The differences in family structures and expectations also play an important role in the nursing profession. It is hard to arrange all-female co-workers’ timetables, as many of them are mothers with similar backgrounds. Male nurses are the coordinators in this form of operation management*.

(Participant #11)

It is worth noting that male nurses’ unique position in the nursing profession is vital, as their knowledge, skills, and characteristics are as necessary as those of their female co-workers. A previous study [10] has also suggested that female scientists often have to give up their career development and turn down promotions due to family responsibilities. Therefore, establishing a gender balance in the nursing profession that accommodates the social expectations of men and women is necessary.

#### 3.1.2. Male Patient Caring Services 

Although both male and female nurses and medical professionals are well-trained for many medical treatments and services, some patients tend to ask for nurses and medical professionals of the same gender (i.e., male patients request male nurses and medical professionals). Unlike westernized societies, in which consideration of gender is often a less important issue, Asian regions value tradition, customs, and religion. Due to social and cultural biases and considerations, many patients in Asia tend to ask for medical professionals of the same gender. However, as nursing is a female-dominated profession, demands for male nurses in the East Asian region cannot always be met. All participants expressed their ideas about how gender is an important consideration in East Asian hospitals and medical facilities due to social and cultural biases. One discussed male patient behavior from a religious perspective:

*In some religious practices, gender is a sensitive topic. Male doctors and nurses cannot touch female patients. If there is a demand, why not provide male human resources? However, due to the occupational bias of male nurses, young boys tend not to join nursing schools. That’s why nursing schools should coordinate with different secondary schools, partnered hospitals, community centers, and elsewhere when making promotions*.

(Participant #18)

Besides religious considerations, some participants suggested that male patients often refuse treatment and services from female nurses and medical professionals due to social, cultural, and personal considerations. One participant stated that female nurses could not change male adult diapers or wash their bodies due to personal considerations about gender:

*Some male patients refuse to take off clothing in front of female professionals, and some female patients refuse to do so in front of male professionals. Patients have the right to request to be treated by a professional of the same gender; we can ask social care providers to accommodate this. Still, some treatment and services must be provided by male nurses. Therefore, we need to train some male nurses*.

(Participant #17)

Due to various internal and external elements, male nurse positions and roles are important in many Asian regions [59,60]. Many participants shared their experiences and their lived stories about working as male nurses and male nursing educators, to motivate potential male nurses who are considering entering the field [6].

#### 3.1.3. Male Frontline Nurses in the Profession

Participants expressed the way in which their gender roles as male nurses in frontline teams increased their interest in encouraging the next generation to join the nursing education profession [61]. Currently, most nursing education faculty members are female nursing practitioners with frontline and administrative experience. However, due to gender issues, the number of male nurses is limited. As a result, there is a human resource shortage of male nursing educators who can share feedback on the operations and perspectives of male nurses in frontline teams. In fact, although both male and female nurses have similar backgrounds and experience of similar working environments, some social and cultural biases and forms of discrimination can only be expressed by particular groups of people (e.g., male nurses). All participants suggested that male nurses and medical professionals are expected to carry out some physical duties, but the experiences were meaningful:

*There are different types of work for nurses in the emergency unit. There are many obese patients, injured patients who can’t move, elderly patients who need extra help. These are all physical forms of work and we expect male nurses and professionals to carry them out. We have social care providers, but male nurses are required in the operation room*.

(Participant #15)

Besides this physical work and frontline operations, the existence and positions of male nurses in the public health field can encourage prospective male nursing students to enter the profession [62]. In fact, due to social and cultural biases, male nurses are the minority in the profession, with gender-oriented biases held toward male professionals. Therefore, positive modeling [18,29] of male nurses can encourage motivation and reduce social biases from members of the general public, as one participant said:

*I believe modelling is key in the nursing profession and nursing education. I learned a lot of knowledge and practices from my placement supervisor, my co-workers, my workplace, and now my school. We use our lives to influence other lives. I use my male nursing modelling and experience to influence other potential male nurses*

(Participant #13)

### 3.2. Respect

Many participants expressed the way in which their nursing practices, at both the hospital level and at the nursing school level, is meaningful and supportive; male nurses are particularly supportive. The respect, support, encouragement, and understanding from patients, family members, patients’ families, members of the general public, school leaders, and even policymakers are important elements in the field of nursing [2]. All participants stated that this encouragement is priceless and supports them in continuing with their teaching positions as male nursing educators at the nursing school level. One participant stated:

*I can solve some problems or male patients’ concerns because of my gender. Also, many students and school professionals love us because of our special roles. When my patients leave the hospital, satisfaction and happiness are priceless. I want to share and continue this love with the next generation*.

(Participant #5)

#### 3.2.1. From Parents and Students 

All participants were faculty members in a school of nursing at a university. Every year, the participants are required to host recruiting seminars and university admission fairs at community centers, secondary schools, and colleges. As male nursing educators, they serve as positive encouragement for many potential students to join the nursing profession, particularly prospective male nursing students [2]. In many societies, parents and students understand and accept the population of male nurses in the public health environment. Several participants shared their remarkable experiences at university admission fairs:

*Many students and parents did not know that male students can join nursing schools. I described my previous experience and told some lived stories to them. All agreed that male nurses are important professionals in hospitals. Although I cannot promise that they will, many students expressed the intention of joining the field*.

(Participant #10)

Besides the way in which male nursing educators serve as role models, many also brought their own nursing students to the admission fairs. Many participants suggested that male nursing students serve as encouragement for prospective students, which allowed those prospective students to ask questions and for placement ideas [26,31,63]. The nursing profession is still a female-dominated profession, due to current social and cultural biases in East Asian communities. In other words, most parents and students do not understand the structure and experiences of male nurses. Therefore, students sharing their experiences can increase interest in (and background understanding of) male nurses, particularly when secondary school alumni participate [61,64]. One participating educator shared his experience of how he coordinated with five male and eight female nursing students to attend an admissions fair:

*I like to bring my students to recruitment fairs. If they can go back to their secondary school, it is meaningful, as they understand the students’ backgrounds. Many received positive messages from their previous counsellors and teachers. We received respect from both parents and students, as we are doing something meaningful for society*.

(Participant #9)

In short, many participants described the way in which working as a male nursing educator is not only a job, but a lifelong and life-changing form of career development [61,65]. In fact, many advocated that working in administrative teams in hospitals allow them to obtain career promotions and enhancements. However, all believed that their goals, interests, and wishes were their first priorities for their teaching career development and pathways.

#### 3.2.2. Social and Cultural Misunderstandings and Concerns

Unlike westernized communities, in which there tends to be better gender equality in working environments, gender considerations are one of the limitations regarding career choices and development in the East Asian region, particularly in nursing and social care professions. Many participants suggested that members of the general public believe nursing is an assisting profession instead of a managing industry. In other words, nurses at the clinical level usually do not have the right to make decisions and perform other management-related tasks, due to the nature of the position and their responsibilities [65,66,67]. However, working as male nursing educators may promote and reduce social and cultural misunderstandings such as these, as well as other concerns held by the general public [6,61]. Furthermore, all-male nursing educators believe they work as models to encourage social equality in the male nursing profession. One participant stated:

*Most general public members believe nursing is only for women and men should not join the profession. But my role, as a model for the profession, is to destroy this social and cultural bias. Nursing is a meaningful profession that welcomes both male and female workers. Over the years, we have gained a lot of respect from the government and people in our communities, as both frontline nurses and nursing educators, particularly during crises*.

(Participant #2)

A similar category of feedback regarding male modeling and working responsibilities that could influence the current biases within the nursing profession was captured [6,61]. Many participants had decided to continue working in their teaching positions, as they wanted to transform the social and cultural biases about male professionals:

*I can go back to the hospital for a senior director’s position, but I want to work in the school, as I am a male educator who can positively model the profession to other male and female students at the nursing school, as well as community members in Asia. Many believe men should not work in the nursing profession, which is not true. I always use myself as an example to destroy this bias*.

(Participant #7)

### 3.3. The Promotion of Health 

The promotion of health, health education, and sexual education are some of the best ways to protect individuals from unhealthy lifestyles, unsafe behaviors, negative activities, and bad habits. In terms of general health recommendations, such as exercise, eating habits, and positive lifestyles, professionals in the field of public health often host conferences and hold meetings and seminars at both micro and macro levels [31]. However, a previous study indicates that, for some sensitive topics, such as sexual education, sexual health, and drinking behaviors, it is important to locate the appropriate personnel. For example, male medical professionals can promote male sexual health knowledge and sharing. All participants stated that they need to transfer men’s sexual health knowledge via workshops and conferences to local male residents, as such knowledge cannot be widely distributed via television, posters, radio, and social media, due to social and cultural considerations.

*School health professionals and public health professionals coordinate to promote health-related messages to local communities. Men and women can work with general health promotions, female professionals usually manage female sexual health promotions and health plans, and male professionals, like me, handle men’s health promotions. Some forms of sexual health knowledge are social taboos in this region*.

(Participant #1)

#### The Promotion of Male Health

All participants held responsibilities regarding health promotions and plans as professionals and leaders at the Department of Health, Council District Center, the Department of Social Welfare, local community centers, social caring centers, and public health sectors. In fact, all participants expressed that gendered-oriented education surrounding sexual health, LGBT issues, and underage sexual problems are vital in all societies, including in Asian regions. However, due to social and cultural taboos and biases, only a few conferences and workshops on these topics can be hosted [68,69,70]. For example, several participants shared the way in which local secondary schools usually invited them to discuss men’s sexual health with male senior high school students:

*There are moral, government, and biology classes at secondary school, but teachers may not be experts on teaching their students about sexual health, so we coordinated with local secondary school teachers to deliver courses on this. Female nursing educators also provide instructions to girls at the schools*.

(Participant #8)

Many participants also engaged with community centers in terms of sexual health and adult health promotions and plans. For example, many have hosted annual middle-aged and senior men’s health conferences and workshops at community centers:

*Our team hosts men’s health workshops for many middle-aged and elderly male residents about benign prostatic hyperplasia, erectile dysfunction, self-testicular exams, and male mental health problems. Such topics cannot be shared by female professionals. The roles of male nursing educators are vital in this case*.

(Participant #12)

LGBT issues have always existed, but are largely ignored in most Asian countries, due to social and cultural taboos and biases. In order to promote and provide help to these groups of minorities, more than half of the participants had engaged with local LGBT social care associations to host outreach services, particularly regarding HIV and gay men’s services [71], based on their gender and expertise. One participant stated:

*My team and my male nursing students like to provide health promotion services and volunteer with local LGBT associations. LGBT people like to share their ideas with people of the same gender or sexual orientation. I like to use my gender and my experience to help LGBT youths and adults experiencing difficulties. It also encourages a connection with my school and local centers*.

(Participant #14)

In short, male nursing educator gender roles encourage their career development and sense of belonging, as communities and residents need their presence and services to obtain better men’s health promotions. Previous studies [31] have indicated that men and sexual minorities tend to prefer to share their sexual problems with people they trust, particularly professionals of the same gender. Therefore, besides providing traditional teaching and learning practices in nursing schools, the participating nursing educators also serve as health promotion specialists, alongside other health and social care professionals.

## 4. Conclusions

To the best of the knowledge, this is one of the first nursing studies that is based on the understanding, sharing, lived stories, and lived experience of male nursing educators who are currently working at one of the nursing schools in the East Asian region. Although there are no limitations and laws to prohibit male nursing educators from joining the nursing education profession, the general public in the East Asian region tended to believe the nursing profession is a female-oriented profession. Therefore, the results of this study captured the life-long career decisions, sense-making processes, and the reasons why these groups of male nursing educators decided to invest their career development in the field of nursing education. 

Based on the theoretical framework (i.e., Social Cognitive Career Theory), it is worth noting that the personal goals and achievements of their career satisfactions took the important roles [10,16,20,72]. Although the general public may not agree with their career decisions due to the gender and social biases, the participants continued to contribute their energy and knowledge for health and social caring professions. 

## 5. Limitations, Future Research Directions, and Implications 

### 5.1. Limitations and Future Research Directions

Every research study has its limitations. Three limitations have been found in this study. First, the research could only cover the participants from China, Japan, South Korea, Taiwan, and Malaysia. However, male nursing educators in Hong Kong, Macau, and Singapore may increase the content of the study as well. Therefore, future research studies can expand the directions and geographic regions to other East Asian countries. 

Second, the current study only covered feedback and interview information from 18 participants. Due to the limited population (i.e., male nursing educators), the researcher could only recruit the above participants. Future research studies and researchers can expand the population to a larger population and participant backgrounds in order to increase the details and contents of the study. 

Third, the research study only covered male nursing educators. However, other health and social caring occupations, such as counselors and social workers, also face similar social biases and discriminations due to their mental and physical conditions. Therefore, further research studies can cover these groups of professionals in order to enhance social justice and equalities in the current health and social caring professions. 

### 5.2. Implications 

Based on the results, there are two potential recommendations. First, the human resources shortage in the field of nursing education is vital. Under the lends of SCCT, this study can work as a blueprint for policymakers, hospital leaders, government agencies, human resources professionals, nursing school leaders, and researchers to reform and upgrade their current policies and regulations for both nursing educators and frontline nursing professionals, regardless of their gender. Although this study mainly captured the feedback and lived stories from male nursing professionals and nursing educators in the East Asian region, professionals from a similar region and country can apply the outcomes to their working environment for better development. 

Second, the outcomes of this study also captured some lived stories and opinions from the perspectives of male nursing educators in the East Asian region. The participants shared numbers of social biases and potential recommendations to the policymakers and government professionals for further improvement. Therefore, with a similar reflection from the future research directions, this study can contribute to the areas of social justice and equalities in the current health and social caring professions. 

## Figures and Tables

**Table 1:** Themes and subthemes.

Name	Region	Highest Degree	Years of Experience in Both Professions
Participant #1	South Korea	Doctorate	20+
Participant #2	South Korea	Doctorate	20+
Participant #3	South Korea	Doctorate	20+
Participant #4	Japan	Doctorate	20+
Participant #5	Japan	Doctorate	25+
Participant #6	Japan	Doctorate	25+
Participant #7	Taiwan	Doctorate	25+
Participant #8	Taiwan	Doctorate	25+
Participant #9	Taiwan	Doctorate	25+
Participant #10	Taiwan	Master	20+
Participant #11	Taiwan	Master	20+
Participant #12	China	Doctorate	25+
Participant #13	China	Master	20+
Participant #14	China	Master	20+
Participant #15	China	Master	20+
Participant #16	Malaysia	Doctorate	25+
Participant #17	Malaysia	Doctorate	25+
Participant #18	Malaysia	Master	20+

**Table 2:** Themes and subthemes.

Themes and Subthemes		
**3.1.**	**Gendered-Oriented Knowledge, Teaching, and Learning**	
	3.1.1.	Male-Oriented Knowledge and Skills in Operation Management
	3.1.2.	Male Patient Caring Services
	3.1.3.	Male Frontline Nurses in the Profession
**3.2.**	**Respect**	
	3.2.1.	From Parents and Students
	3.2.2.	Social and Cultural Misunderstandings and Concerns
**3.3.**	**The Health Promotion**	
		The Promotion of Male Health

## Appendix A

First-Round Interview Protocol

1) Why do you want to become a male nurse? Please tell me more.
2) May you share some stories about your frontline experiences as a male nurse? Please tell me more.
3) Do you enjoy being a male frontline nurse? Please tell me more.
4) What makes or made you stay in the nursing profession? Please tell me more.
5) Follow-up questions.

Second-Round Interview Protocol

1) Can you tell me the reasons and motivations why do you want to switch your career pathway from frontline nurse to nursing education? Please tell me more.
2) Do you enjoy the switching? Please tell me more.
3) Why switch if you like the frontline experiences? Please tell me more.
4) As a male professional, would your gender positive and negatively impact the switching? How and why?
5) Follow-up questions.

Third-Round Interview Protocol

1) Can you tell me about your experiences as male nursing educators? Anything.
2) How would you describe your experiences and ideas about being a male nursing educator? Please tell me more.
3) Do you enjoy your current position(s)? Why and how?
4) What are the reasons and motivations make/made you stay in the profession? Please tell me more.
5) Follow-up questions.
